# Neuromorphic Spiking Neural Networks and Their Memristor-CMOS Hardware Implementations

**DOI:** 10.3390/ma12172745

**Published:** 2019-08-27

**Authors:** Luis A. Camuñas-Mesa, Bernabé Linares-Barranco, Teresa Serrano-Gotarredona

**Affiliations:** Instituto de Microelectrónica de Sevilla (IMSE-CNM), CSIC and Universidad de Sevilla, 41092 Sevilla, Spain

**Keywords:** neuromorphic systems, spiking neural networks, memristors, spike-timing-dependent plasticity

## Abstract

Inspired by biology, neuromorphic systems have been trying to emulate the human brain for decades, taking advantage of its massive parallelism and sparse information coding. Recently, several large-scale hardware projects have demonstrated the outstanding capabilities of this paradigm for applications related to sensory information processing. These systems allow for the implementation of massive neural networks with millions of neurons and billions of synapses. However, the realization of learning strategies in these systems consumes an important proportion of resources in terms of area and power. The recent development of nanoscale memristors that can be integrated with Complementary Metal–Oxide–Semiconductor (CMOS) technology opens a very promising solution to emulate the behavior of biological synapses. Therefore, hybrid memristor-CMOS approaches have been proposed to implement large-scale neural networks with learning capabilities, offering a scalable and lower-cost alternative to existing CMOS systems.

## 1. Introduction

The outstanding evolution of computers during the last 50 years has been based on the architecture proposed by Von Neumann in the 1940s [1]. In this model of stored-programme computer, data storage and processing are two independent tasks performed in separated areas with a high need of data communication between them. With the development of integrated circuits, Gordon Moore predicted in the 1960s that the number of transistors in an integrated circuit would double every 18 to 24 months [2]. This exponential evolution allowed for the development of more efficient computing systems, with increasing processing speed and decreasing power consumption. However, even the current technologies for semiconductor manufacturing are reaching the limits of Moore’s law [3], so different solutions have been proposed to keep the future evolution of processing systems [4]. Two different strategies suggest the development of new processing paradigms and novel devices beyond conventional Complementary Metal–Oxide–Semiconductor (CMOS) technologies.

In parallel with the development of computing platforms, in the 1960s some researchers used the emerging electronic technologies as a mechanism for modeling neural systems, from individual neurons [5,6,7,8,9,10] to more complex networks [11]. The increasing understanding of the structure and fundamental principles of behavior of the human brain revealed a very different processing paradigm from the traditional computer architecture with a much better performance. Even when comparing with current supercomputers which excel at speed and precision, the human brain is still much more powerful when dealing with novelty, complexity and ambiguity for practical tasks like visual recognition and motion control, while presenting a negligible power consumption around 20W [12]. This comparison between conventional computers and the brain led to the emergence of neuromorphic computing. The term neuromorphic engineering was first coined by Carver Mead to refer to developing microelectronic information processing systems mimicking the operation of their biological counterparts [13,14]. During the 1980s, Carver Mead highlighted the analogy between the physics in biological neurons and the behavior of transistors in sub-threshold regime [13,14], developing neural networks based on analog circuits; leading to the implementation of the first silicon retinas [15] and proposing a new computing paradigm where data and processing tasks are performed by indivisible entities, taking inspiration from biological neural systems. Along the years, the neuromorphic engineering field has broaden its inspiration. Today’s neuromorphic computing engineers not only try to mimic the highly parallel architecture of biological brains and the use of in-memory computing architectures as a way of improving the speed and energy performance, but also have deeply studied the signal information encoding, computational principles and learning paradigms that enable even simple biological brains with admiring performance in the interaction and adaptation to complex and unexpected environments with high reaction speeds and minimal power consumption despite relying on very simple and highly unreliable computation units [16].

Alternatively, many novel beyond-CMOS technologies have been proposed to overcome the limits of Moore’s law. One of the most promising available devices is the nanoscale memristor. The memristor was first described theoretically by Chua in the 1970s as the fourth passive element establishing a relationship between electric charge and magnetic flux [17]. Much later in 2008, a team at HP Labs claimed to have found Chua’s memristor experimentally based on a thin film of titanium oxide [18]. This 2-terminal device behaves as a variable resistor whose value can be modifed by applying certain voltages or currents. The most common structure for this device is a union metal-dielectric(s)-metal, where the dielectric layer can be as thin as a few nanometers. The application of electric fields and controlled currents across the dielectric produces an alteration of its resistance by growing a filament or other mechanisms like barrier modulation. Currently available memristors are mostly binary devices, as they can switch between two resistance values: HRS (High-Resistance State) and LRS (Low-Resistance State) [19]. Since the appearance of the memristors, many logic families based on memristors for digital computation have been proposed [20,21], their potential as digital long-term non-volatile memory technology has also been demonstrated [22,23,24,25], and their use as biosensing devices looks also promising [26]. In the field of neuromorphic engineering, the memristors have attracted a special interest due to its particular plasticity behaviour which ressembles the adaptation rules observed in biological synapses. Memristors can adapt and change its behaviour over time in response to different stimulation patterns as it happens in the human brain. In particular, it has been demonstrated that if stimulated with pulse-trains simulating the input from spiking neurons, memristors may exhibit a biologically inspired learning rule [27,28,29,30] resembling the spike-timing-dependent plasticity (STDP) observed in biological neurons [31,32,33,34,35,36]. Hence, memristors have been considered as artificial inorganic synapses.

In this paper, we analyze the current trend towards using memristors over CMOS platforms to implement neuromorphic systems, demonstrating a new paradigm which overcomes current limitations in conventional processing systems. In Section 2, we give a general overview of the basis of neuromorphic computing, while in Section 3 we review the main large-scale CMOS hardware implementations of neuromorphic systems. In Section 4, we describe proposed hybrid Memristor-CMOS approaches, while in Section 5 we emphasize the suitability of this strategy to implement learning algorithms in neural systems. Finally, in Section 6 we give our future perspective for this field.

## 2. Neuromorphic Computing

As already stated, neuromorphic computing systems take inspiration on the architecture, the technology and the computational principles of biological brains. Morphologically, the human brain is composed of approximately $10^{11}$ elementary processing units called neurons, massively interconnected by plastic adaptable interconnections called synapses. Each neuron connects approximately to $10^{3}$–10${}^{4}$ other neurons through synaptic connections. The neurons are known to be distributed in layers, and most of the synaptic interconnections are devoted to interconnect neurons belonging to successive layers.

The first computing systems inspired by this structure of biological brains were published in the 1940s–1950s and were called Artificial Neural Networks (ANNs) [37,38]. They appeared as powerful computational tools that proved to solve, by iteratively training algorithms that adapted the strength of the interconnection weights, complex pattern recognition, classification or function estimation problems not amenable to be solved by analytic tools. The first generations of neural networks did not involve any notion of time nor any temporal aspect in the computation.

**Figure 1:**
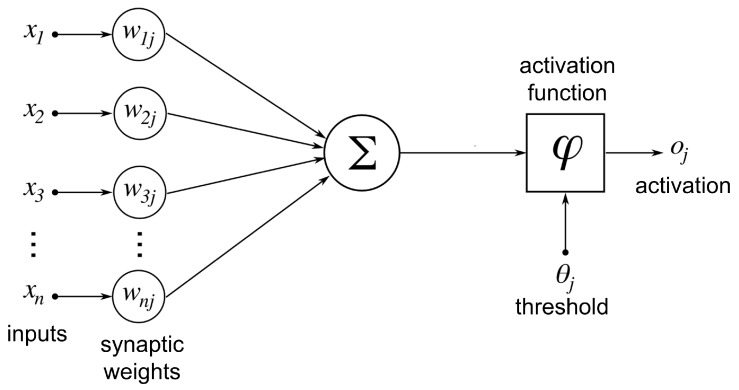
Diagram of an artificial neuron with n inputs with their corresponding synaptic weights. All weighted inputs are added and an activation function controls the generation of the output signal.

Mc Culloch and Pitts, proposed in 1943, one of the first computational models of the biological neurons. Figure 1 illustrates the operation of each proposed neural computational unit. As illustrated in Figure 1, a neuron $N_{j}$ receives inputs from *n* other previous neurons $x_{1},x_{2},\ldots ,x_{n}$. The output of each neuron $x_{1},x_{2},\ldots ,x_{n}$ in the previous layer is multiplied by the corresponding synaptic weight $w_{1j},w_{2j},\ldots ,w_{nj}$, also know as synaptic efficacy. The combined weighted input is transformed mathematically using a certain non-linear transfer function or an activation function φ, generating an output $o_{j}$. In the original Mc Culloch and Pitts’ neural model the activation function was a thresholding gate, giving as neural output a digital signal [37]. This digital output neuron was the core of the first generation of neural networks.

**Figure 2:**
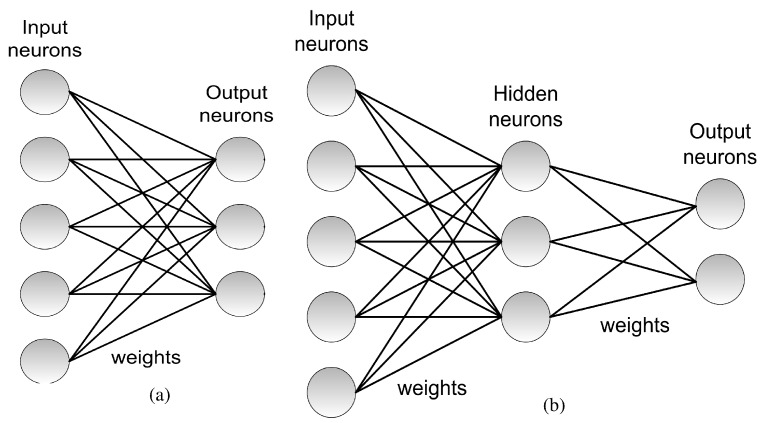
(**a**) Architecture of a single layer perceptron. The architecture consists of a layer on input neurons fully connected to a single layer of output neurons. (**b**) Extension to a multi-layer perceptron including more than one layer of trainable weights. In this example, the network includes 3 layers: input, hidden and output layer. Each connection between two neurons is given by a certain weight.

In 1958, Rosenblatt proposed the perceptron. The architecture of the perceptron is shown in Figure 2a. In Figure 2, the computational units or neurons are represented by circles, interconnected through trainable weights representing the synaptic connections. The original perceptron consisted of a single layer of input neurons fully interconnected in a feedforward way to a layer of output neurons. A learning hebbian rule [39] to adapt the weights was proposed [38]. This single layer perceptron was able to solve only linearly separable problems [40].

In the 1950–60s, a second generation of computational units arose were the thresholding activation function was replaced by a continuous analog valued output like a smooth sigmoid, radial basis function or a continuous piece-wise linear function [41,42]. Recently, the rectifying non-linear activation function, also known as ReLU has become very popular for its better training convergence and its hardware friendly implementation [43]. Furthermore, gradient descent based learning algorithms could be now applied to optimize the network weights. Alternative learning rules were proposed as the delta rule based on the Least Mean Squares (LSM) algorithm published by Widrow [44,45]. This second generation proved to be universal approximators for any analog continuous function, that is, any analog continuous function could be approximated by a network of this type with a single hidden unit [41].

The backpropagation algorithm extended the application of the gradient descent techniques to networks with any number of hidden layers, popularly known as Deep Neural Networks (DNNs) [46,47,48]. Figure 2b illustrates a case with 3 layers: a first layer of input neurons, a second layer of hidden neurons, and a third layer of output neurons, although a general architecture can contain any given number of hidden layers.

The ANN architectures shown in Figure 2a,b are pure feedforward architectures as the signal propagates from input to output in an unidirectional way. Other architectures, known as recurrent neural networks, including feedback connections from upper layers in the architecture to lower layers, have been proposed. The Adaptive Resonance Theory (ART) architectures by Grossberg [49], the Kohonen self-organizing maps [50] or the Hopfield models [51] can be cited among the pioneering ones.

The presented ANNs have been typically developed in software, and trained offline. The training of DNNs requires a vast amount of annotated data to correctly generalize the problem without overfitting [52] and intensive computation resources. However, in recent years, the increase in the computation capabilities of modern computers and the availability of vast amounts of information have made DNN very popular allowing the development of many DNN-based applications [53,54] that use complex architectures like LeNet for handwritten digit recognition [55], Microsoft’s speech recognition system [56] or AlexNet for image recognition [43]. As a consequence we have witnessed the explosion of DNNs and machine learning.

Despite the impressive advances that DNNs have demonstrated in recent years, their performance in terms of efficiency (speed and power consumption) compared with the human brain is still low as it is low their resemblance to the human brain in terms of information coding. In the biological brain, the information is processed in a continuous way in time, not just as a sequence of static frames as DNNs recognition systems do. Furthermore, in conventional DNNs, the output of the different neural layers are computed in a sequential way. Each layer has to wait until the output of the previous layer has been computed to perform its computation, thus introducing a significant recognition delay in the network. On the contrary, biological neurons transmit their information to the next neuronal layers in the form of spikes. Whenever a neuron emits a spike, the spike is transmitted to its afferent connected neurons and processed with just the delay of the synaptic connection. In 1996, Thorpe demonstrated that the human brain was able to recognize a visual familiar object in the time that just one spike propagates through all the layers of the visual cortex [57]. Similar visual processing speeds have been measured in the macaque monkeys by Rolls [58]. These experiments reveal an extremely efficient information coding in the biological brains. In this context, the 3rd generation of neural networks, spiking neural networks (SNNs), aims to bridge the gap between neuroscience and machine learning, using biologically-realistic models of neurons to carry out information coding and computation trying to fully exploit the efficiency in the spatio-temporal signal coding and processing and the corresponding power efficiency observed in the biological brains. SNNs operate using spikes in a similar way as biological neurons do. That way, in addition to the state of the neuron and the synaptic weight, SNNs also incorporate the concept of time into their model of operation. In these neurons, there is no propagation cycle, so each neuron fires an output spike only when its state reaches a certain threshold. Therefore, the information flows in these networks are spike trains which propagate between neurons asynchronously, and temporal correlation between spikes is crucial [41]. Spike trains offer the possibility of exploiting the richness of the temporal information contained in real-world sensory data. This allows SNNs to be applied to solve tasks which dynamically changing information like visual gesture recognition or speech recognition in a more natural way than current conventional (non spiking) artificial intelligent systems do. When dealing with dynamic information (as video sequences), conventional artificial systems perform computations using sequences of static images sampled at a constant periodic time (photogram time in the case of vision). Recognition of dynamic sequences may involve the use of recurrent neural network architectures or the resolution of continuous time differential equations. These computations are quite intensive using conventional framed ANN. However, the use of SNN where computation is driven in a continuous time way naturally and driven only by the occurrence of spikes detecting certain spatio-temporal correlations can be much more advantageous.

Many different coding methods for these spike trains have been proposed. Many authors have proposed to code the activity level of the neurons as the frequency of the firing rate. However, this type of coding does not benefit from the spike sparsity that should characterize SNN processing and thus, it does not enable the corresponding low power communication and computation due to the sparsity of the spike coding. Regarding the fast computation capability expected from SNN, this firing rate coding introduces a latency in the computation of the output firing rate. Furthermore, it is not biologically plausible as evidenced by the experiments of Thorpe [57] and Rolls [58] which demonstrated that the computation of a single cortical area is completed in 10–20 ms while the firing rate of the neurons involved in the computation is below 100 Hz, which does not make possible the computation based on the coding of analog variables in firing rates. However, as discussed by Thorpe et al. [59], there are many other biologically plausible and more efficient coding strategies. Other coding schemes that have been considered are in the timing between spikes [60], in the delay relative to a given synchronization time also known as time to first spike (TFS) [59] encoding, just coding the values in the order of spikes which is known as rank order coding [61], or synchronous detection coding [59].

Regarding the SNN neuron models, there are many neuron models that describe the behaviour of biological neurons with different levels of complexity [5,6,7,8,9,10]. The classic Hodgkin-Huxley model [5] is a 4-th order biophysical model that describes the behaviour of the currents flowing into the neuron ion channels in a biologically realistic way. However, due to its complexity, different 2nd order simplified models have been proposed like the one proposed by FitzHugh and Nagumo [6,7] and the Morris-Lecar model [8], among others. In the last years, the Izhikevich model [10] and the Adaptive Exponential Integrate and Fire (AdEx) model [9] have become very popular for their ability to reproduce a large variety of spiking regimes observed in the biological neurons just by varying a reduced number of model parameters. However, while detailed biophysical models can reproduce electrophisiological activity of biological neurons with great accuracy, they are difficult to analyze computationally and not friendly for hardware implementations. Because of these reasons, for computational purposes simple first-order phenomenological models like the Integrate and Fire model are frequently used.

**Figure 3:**
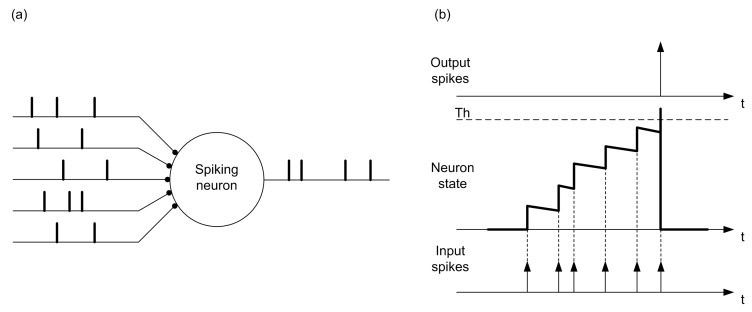
Illustration of the behavior of a leaky integrate-and-fire spiking neuron. (**a**) A spiking neuron receives spikes from several inputs, processes them, and generates output spikes from its output node. (**b**) Temporal evolution of the neuron state while it receives input spikes. When the threshold is reached, it generates an output spike.

**Figure 4:**
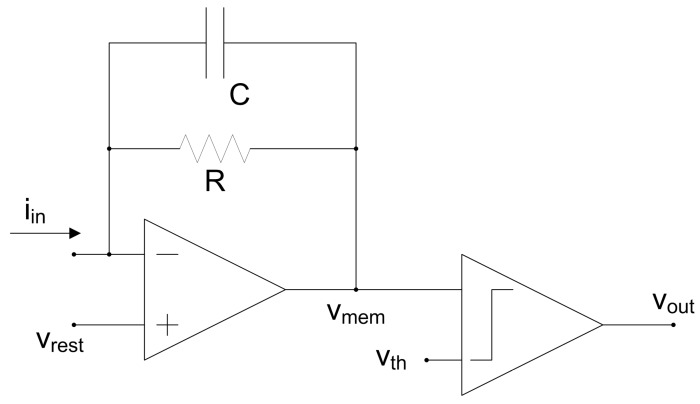
Example of a hardware implementation of an RC leaky integrate-and-fire neuron.

The behavior of a single integrate-and-fire spiking neuron is illustrated in Figure 3. A spiking neuron receives input spikes from several dendrites and sends out spikes from its output axon, as shown in Figure 3a. Every time an input spike arrives, the state of the neuron is updated, and when it reaches the threshold, it generates an output spike and reset its state, as seen in Figure 3b. In this case, spikes are fully characterized by their firing time. In Figure 3, it can be observed that there is a constant slope decay of the membrane potential between two arriving spikes as it is the case of a leaky integrate and fire neuron. Mathematically, a leaky integrate-and-fire neuron can be described as:

(1) $$\begin{array}{c} i_{in}\left(t\right)=\frac{v_{mem}\left(t\right)-v_{rest}}{R}+C\frac{dv_{mem}\left(t\right)}{dt} \end{array}$$

where $v_{mem}\left(t\right)$ represents the membrane potential, $i_{in}\left(t\right)$ the injected current, $v_{rest}$ the resting value of the membrane potential, *C* the equivalent capacitance of the membrane, and *R* the leak resistance. A leaky integrate-and-fire neuron can be easily implemented in hardware following the resistance-capacitance (RC) "text book" concept scheme presented in Figure 4, where an input current $i_{in}$ is integrated in capacitor *C* with leak resistance *R*. The integrated voltage $v_{mem}$ is compared with a reference $v_{th}$, generating an output given by $v_{out}$. Additionally, integrate-and-fire neurons may consider a refractory period that forces a minimum time interval between two consecutive spikes of a neuron. A comprehensive overview of circuit realizations of spiking neurons with different levels of complexity can be found in [62].

In terms of connectivity, the most general type of neural network is fully connected, meaning that each single neuron in layer *i* is connected to all neurons in layer i+1. This scheme applies no limitation to the learning capabilities of the network; however, it presents some difficulties for practical implementations. A very popular way of reducing the amount of interconnections is represented by Convolutional Neural Networks (ConvNets), where each neuron in layer *i* is connected to a subset of neurons in layer i+1 representing a projective field. This receptive field can be represented as a convolutional kernel, with shared weights for each layer [63]. This scheme is inspired by biology, as it has been observed in the visual cortex [64]. In a similar way to the biological visual cortex, this convolutional neural network architecture is commonly used for image processing applications in the earlier more massive parallel feature extraction layers, as it implies an important reduction of the number of connections.

Table 1 (adapted from [65]) contains a comparison of the main distinctive features between ANNs and SNNs. As previously stated, the latency in each computation stage in an ANN is high as the whole computation in each stage has to be completed on the input image to generate the corresponding output. On the contrary, in an SNN processor the computation is performed spike by spike so that, output spikes in a computational layer are generated as soon as enough spikes evidencing the existence of a certain feature has been collected. In that way, the output of a computation stage is a flow of spikes that is almost simultaneous with its input spike flow. This property of SNN systems has been called “pseudo-simultaneity” [65,66]. The latency between the input and output spike flows of a processing SNN convolution layer has been measured to be as low as 155 ns [67]. Regarding the recognition speed, whereas in an ANN the recognition speed is strongly dependent on the computation capabilities of the hardware and the number of total operations to be computed (which is dependent on the system complexity), in an SNN, each input spike is processed in almost real time by the processing hardware and the recognition is performed as soon as there are enough input events that allow the system to take a decision. This recognition speed strongly depends on the input statistics and signal coding schemes as previously discussed. In terms of power consumption, the ANNs power depends on the consumption of the processor and the memory reading and writing operations but for a giving input sampling frequency and size does not depend on the particular visual stimulus. However, in an SNN, the power consumption depends also strongly on the statistics of the stimulus and coding strategies. If efficient coding strategies are used, the system should benefit from the power efficiency of sparse spike representations.

On the negative side, as it has been already pointed out, the addition of the time variable makes SNN neuron models more complex than ANN ones. Also, as the computation of ANN is time-sampled, in each sampling time the algorithmic computation is performed using the available hardware resources that can be time multiplexed by fetching data and storing intermediate variables. However, in true SNN the spikes should be processed as they are generated in real time, requiring parallel hardware resources which cannot be multiplexed. The scaling up of the system can be done by modular expansion of the hardware resources.

However, where SNN should have major advantage is in applications requiring recurrent neural architectures, such as, in recognition of dynamic stimulus. The computation of recurrent connections in ANN requires computationally intensive iterations until convergence is reached, while the convergence of recurrent connections in SNN is almost instantaneous due to their pseudo-simultaneity property.

In terms of accuracy, as it will be discussed in Section 5, the learning methods that have been developed for ANN are not directly applicable to SNN. Although the learning theory of SNN still lacks behind its equivalent methods for ANN, some recent work reports for the same architecture an error increment of only 0.15% for the ImageNet dataset and 0.38% for the CIFAR10 dataset [68]. However, the temporal dependence introduces complexity so that once a SNN has been trained, its accuracy drops if the input temporal coding changes. But it also introduces the potential to recognize dynamic sequences in a more efficient way.

## 3. CMOS Neuromorphic Systems

Simulating SNNs on normal hardware is very computationally-intensive since it requires simulating coupled differential equations of large neuron populations running in parallel. Fully exploiting the coding and computation capabilities of biological brains requires the adequacy of the corresponding hardware platform to the peculiarities of the algorithm at different levels: from signal coding up to high level architectures. At the architectural level, the intrinsic parallelism of neural networks lends to the development of neuromorphic custom parallel hardware resembling the architecture of the biological brain to emulate its computing capabilities [62,69,70]. Furthermore, at the signal level, SNNs are better suited than ANNs for hardware implementation, as neurons are active only when they receive an input spike, reducing power consumption and simplifying computation.

**Figure 5:**
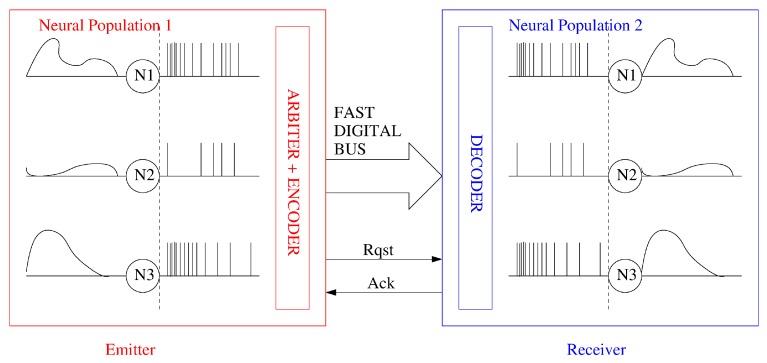
Illustration of two neural populations communicated through a point-to-point AER bus. Each neuron in the emitter population can be virtually connected to every neuron in the receiver population.

One of the major issues when trying to implement in a parallel hardware large arrays of neural populations is the implementation of the synaptic interconnections. In a parallel 2D hardware, the physical wiring does allow to implement connections between just neighbouring neurons, while the biological neurons are distributed in 3D and massively interconnected among populations. Address-Event-Representation (AER) [71] is an asynchronous communication protocol that was conceived to massively interconnect neuron populations that can be located in the same or different chips as a ‘virtual wiring’ system. Figure 5 illustrates two neural populations communicated through an AER bus. In the particular case of this figure, neurons in the emitter population code their activity as a density of output pulses which is proportional to their activation level. However, the AER communication scheme can be applied to any type of pulse signal encoding [59]. Whenever a neuron in the emitter population generates a spike, it codes its physical coordinates (x,y) or address in a digital word in a fast digital bus and activates an asynchronous request (Rqst) signal. The coded address is sent through the fast digital bus to the receiver population. Upon reception of an active request, the receiver decodes the arriving neuron address and activates the acknowledge (Ack) signal. The received pulse can be sent to the corresponding neuron where the original activity of the sending neurons can be reproduced (as illustrated in Figure 5) or to a group of virtually connected neurons in the receiving population implementing a projection field [72]. The high-speed of the inter-population digital bus (in the order of nanoseconds) compared to the inter spike interval of biological neurons (in the order of milliseconds) allows to multiplex the connections of a million neurons in a shared time-multiplexed digital bus. Most of the developed large-scale CMOS neuromorphic computing platforms make use of this AER communication protocol. As neuromorphic systems have scaled up in size and architectural complexity, many variations of the original point-to-point AER communication scheme [71,73,74] have been proposed trying to improve the overall system communication bandwidth. The broadcast-mesh-AER [75,76,77] proposes a generic approach to interconnect a mesh of AER devices using a global mapper and interconnecting the devices in a chain architecture. The pre-structured hierarchical AER approach [78] uses the knowledge of the network topology to interconnect AER devices through different AER links. Mappers can be used in every link, however, once the AER devices have been physically interconnected the changes in the configuration are limited. The Hierarchical-Fractal AER [79] proposes different levels of interconnection by adding address bits at higher level based on the idea that the traffic of spikes is going to be more intense at a local level. The router-mesh AER [80] proposes to avoid an external mapper by placing a router with a mapping table inside every AER module taking ideas from traditional NoC topologies [81]. The multicasting-mesh AER approach [82] proposes a simplification of the router-mesh AER by employing routing tables that contain only information of the connectivity between modules instead of allowing full neuron to neuron connectivity programming. Another approach developed to allow programmable interconnections inside the same chip or at wafer scale has been to implement massive programmable cross-point interconnects to configure the network topology [83] and including off-wafer rerouting for longer range interconnects [84]. Recently, the Hierarchical Routing AER has been proposed that establishes different hierarchical levels of nested AER links where each link has a dynamically reconfigurable synaptic routing table which allows programmable connectivity of the neurons without restriction on the spatial range of connectivity [85]. Moradi et al. have proposed a mixed-mode hierarchical-mesh routing scheme that exploits a clustered connectivity structure to reduce memory requirements and get a balance among memory overhead and reconfigurability [86].

The above mentioned spike routing schemes have allowed the implementation of highly parallel massively interconnected spiking neural networks and the multichip integration of SNN hardware devoted to realize different specific parts of the cognitive function including integration of spike-based sensors and neural processors.

**Figure 6:**
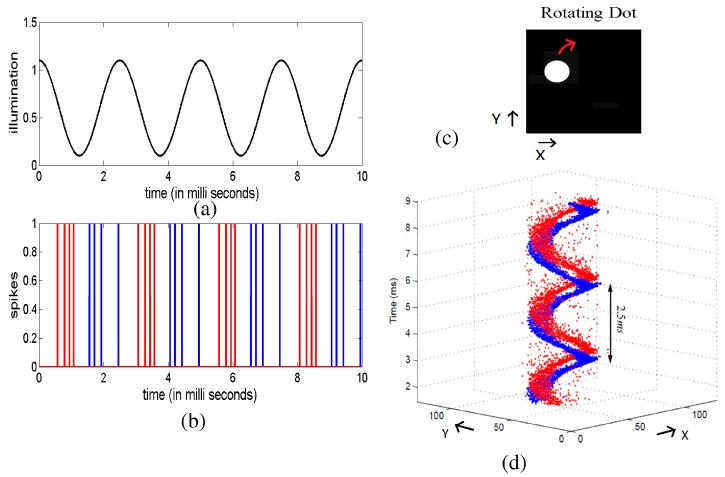
Illustration of the operation of a Dynamic Vision Sensor. (**a**,**b**) illustrate the operation a DVS pixel. (**a**) plots the illumination inpinging on a pixel that varies as a sinusoidal waveform along time with period 2.5 ms, and (**b**) illustrates the output spikes generated by the corresponding DVS pixel. The blue traces correspond to positive output spikes which are generated when the illumination increases, while the red traces illustrate the negative signed spikes generated by an illumination decreasing over time. (**d**) illustrates real measurements of the response of a DVS when observing a white rotating dot on a black background rotating with a 2.5 ms period, as shown in (**c**).

CMOS spike-based vision sensors have been developed since the very beginning of the neuromorphic engineering field [15]. Since then, a variety of AER visual sensors can be found in the literature that use different approaches to encode the luminance such as simple luminance to frequency transformation sensors [87], Time-to-First-Spike (TFS) coding sensors [88,89,90,91], foveated sensors [92,93], sensors encoding the spatial contrast [94,95], spatial and temporal filtering sensors that adapt to illumination and spatio-temporal contrast [96] and temporal transient detectors [97,98,99,100,101,102,103,104]. Among them, the temporal transient detectors also know as Dynamic Vision Sensors (DVSs) have recently become very popular. They produce as output a stream of asynchronous events where each pixel codes the temporal variation of the illumination inpinging on the pixel. Figure 6 illustrates the operation of a DVS sensor. One of the advantages of this sensor is that it codes the information in a compressive way sending only spikes when there is a relevant change in the illumination and thus removing the static background features of the scene from the moving object. Another advantage is that all the exact spatio-temporal information of the object is preserved with a reported precision in the spiking times of the order of 10μs. This converts these sensors in ideal candidates for high-speed processing and recognition systems. Several companies are nowadays making an effort to develop commercial prototypes of high-resolution DVS cameras: iniVation, Insightness, Samsung [105], CelePixel [106], and Prophesee, aiming to develop high-speed autonomous intelligent vision systems. Other types of spiking sensors have been developed such as cochleas [107,108,109] and tactile sensors [110,111] following similar principles of encoding the sensed signal relative changes as a flow of neural spikes, thus, generating a compressed information.

Regarding the neuromorphic hardware for processing, it should be distinguished between the hardware implementing specific functionalities of the cognitive function and general purpose SNN hardware platforms intended for emulating massive neural arrays. Among the specific functional neuromorphic circuits, researchers have developed SNN neuromorphic chips implementing computational primitives and operations performed in the brain such as:

- Winner-Take-All (WTA) is a brain inspired mechanism implemented by inhibitory interactions between neurons in a population that compete to inhibit each other. The result is that the neuron in the population receiving the highest input remains active while silencing the output of the rest of the neurons. Hardware modules of spiking Winner-take-all networks have been reported [112].
- Spiking Convolutional Networks (ConvNets): neural networks implementing in real time the behaviour of the feature extraction layers of the cortex region have been implemented in hardware [113,114,115].
- Hardware implementations of spiking neural networks for saliency maps detection have been proposed as emulators of brain attention mechanisms [116].
- Spiking Liquid State Machines have also been implemented for recognition of sequential patterns such as speech recognition tasks [117,118].

The specific SNN neuromorphic chips can be combined in a modular and scalable way [78] to achieve optimum performance in terms of complexity, speed, and power consumption depending on the specific application. However, the current evolution of hardware neuromorphic platforms tends to large-scale modular computing systems with increasing numbers of neurons and synapses [62,119] that are meant to be easily reconfigurable for different applications. Some of the most remarkable large-scale neuromorphic systems developed until the present are:

- The IBM TrueNorth chip is based upon distributed digital neural models aimed at real-time cognitive applications [120].
- The Stanford NeuroGrid uses real-time sub-threshold analogue neural circuits [121]. It has been recently reversioned with the Braindrop chip prototype [122] which is a single core planned to be part of the 1-million-neuron Brain Storm System [123].
- The Heidelberg BrainScaleS system uses wafer-scale above threshold analogue neural circuits running 10,000 times faster than biological real time aimed at understanding biological systems, and in particular, long-term learning [124].
- The Manchester SpiNNaker is a real-time digital many-core system that implements neural and synapse models in software running on small embedded processors, again primarily aimed at modelling biological nervous systems [125].
- The Intel Loihi chip consists of a mesh of 128 neuromorphic cores with an integrated learning engine on-chip [126].
- The Darwin Neural Processing Unit is a hardware co-processor with digital logic specifically designed for resource-constrained embedded applications [127].
- The ROLLS chip was developed at ETHZ-INI including 256 neurons and 128 k on-line learning synapses [128]. Recently, it has been updated to the Dynamic Neuromorphic Asynchronous Processor (DYNAPs) with 1 K neurons and 64 k on-line learning synapses [86].
- A digital realization of a neuromorphic chip (ODIN) containing 256 neurons and 64 K 4-bit synapses exhibiting a spike-driven synaptic plasticity in FDSOI 28 nm technology has recently been developed in the University of Leuven [129].

A comparison of the main features of these generic neuromorphic systems and the human brain is shown in Table 2. In general, these systems are based on a processing chip which is part of a multi-chip board (or wafer for BrainScaleS), and in some cases these boards can be assembled in multi-board racks, scaling up more and more the size of the implemented network. Some of the most recent approaches have not reported yet such multi-chip platforms.

## 4. Hybrid Memristor-CMOS Systems

As was mentioned in Section 1, progress in silicon technologies is reaching physical limitations which are causing the end of Moore’s law, and traditional Von Neumann computing architectures are reaching scalability limits in terms of computing speed and power consumption. Novel brain inspired architectures have emerged as alternative computing platforms specially suitable for cognitive tasks that require the processing of massively parallel data. As already stated in Section 3, one of the main bottlenecks of the CMOS implementation of these neuromorphic parallel architectures is the physical implementation of the massive synaptic interconnections among neurons and the synaptic adaptability. The implementation of adaptable synaptic connections in CMOS technology requires the use of large amount of circuitry for analog memory or digital memory blocks that are costly in terms of area and energy requirements. Furthermore, learning rules to update these synaptic memory devices have to be implemented. The interest in developing a compact adaptable device obeying biological learning rules to implement the synaptic connections has motivated the investigation on alternative nanotechnologies to complement the CMOS technology in this regard. Memristive devices are novel two terminal devices able to change their conductance as a function of the voltage/current applied to their terminals that were predicted in 1971 by Chua based on circuit theory reasoning [17] and whose existence was experimentally demonstrated in nanomaterials devices much later in 2008 [18]. Different materials with different conductance switching mechanisms have been proposed [130] such as Phase-Change-Memory (PCM) [131], Conductive Bridge Memory (CBRAM) [132], Ferroelectric Memories (FeRAM) [133], Redox-based resistive switching Memories (ReRAM) [134], or organic memristive devices (OMD) [135,136,137,138,139]. Each of them presents different characteristics in terms of compactness, reliability, endurance, memory retention term, programmable states, and energy efficiency [69,140].

These devices present some properties specially valuable as electronic synaptic elements [141]:

- Memristors can be scaled down to feature sizes below 10 nm.
- They can retain memory states for years.
- They can switch with nanosecond timescales.
- They undergo spike-based learning in real time under biologically inspired learning rules as Spike-Time-Dependent Plasticity (STDP) [31,32,34,35,36].

**Figure 7:**
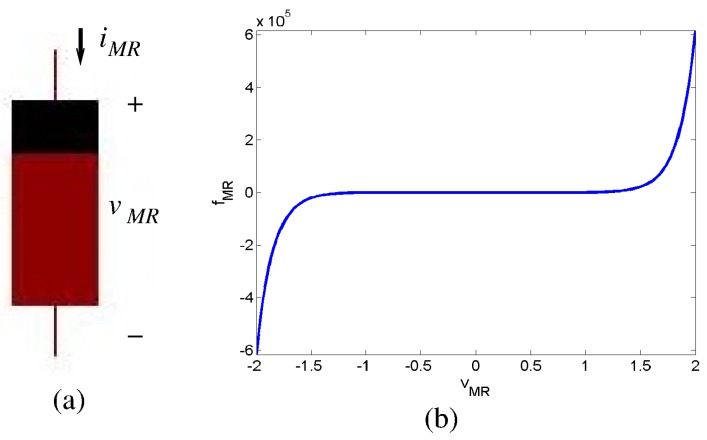
(**a**) Memristor symbol and (**b**) typical thresholded memristive adaptation curve

The characteristic i/v equations of a memristive element can be approximated by:

(2) $$\begin{array}{c} \begin{array}{c} i_{MR}=G\left(w,v_{MR}\right)v_{MR}\\ dw/dt=f_{MR}\left(w,v_{MR}\right) \end{array} \end{array}$$

where $i_{MR}$, $v_{MR}$ are the current and the voltage drop at the terminal devices, respectively (as shown in Figure 7a, $G(w,v_{MR})$ is the conductance of the device that changes as function of the applied voltage (supposing a voltage or flux controlled device model [142]), and *w* is some physical parametric characteristic whose change is typically governed by a nonlinear function $f_{MR}$ of the applied voltage including a threshold barrier. A typical $f_{MR}$ observed in memristive devices [142] can be mathematically approximated by [28,29,30,143]

(3) $$\begin{array}{c} f_{MR}=\left\{\begin{array}{cc} I_{o}*sign\left(v_{MR}\right)\left(e^{|v_{MR}|/v_{o}}-e^{v_{TH}/v_{o}}\right) & if\;|v_{MR}|>v_{Th}\\ 0 & otherwise \end{array}\right. \end{array}$$

Figure 7b depicts the typical non-linear memristive adaptation curve $f_{MR}$.

**Figure 8:**
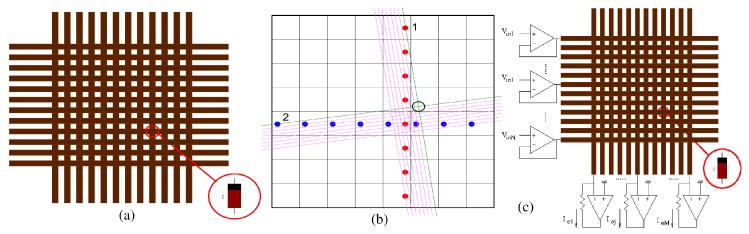
Illustration of the proposed hybrid CMOS/memristive CMOL architecture. (**a**) Memristive devices fabricated in the cross-points of a crossbar array and (**b**) proposed CMOL architecture. (**c**) Neuromorphic architecture composed of CMOS neurons connected to a crossbar array of memristors.

According to Equations (2) and (3), when a voltage higher than $v_{TH}$ is applied between the terminals of a voltage-controlled memristor, its resistance changes. This property has been used to adapt supervisely the weights of simple perceptron networks [38] by applying voltage pulses controlled by some error function to memristive devices. The performance of correct categorization has been experimentally demonstrated [144,145,146]. Although these novel memristive devices open very promising alternatives for electronic technologies, they are still far from the maturity reached by CMOS sytems during the last decades. Instead, they are very promising technologies for being integrated in 3D with CMOS technology providing a high-density memory closely tight to computational units, thus overcoming the limitations of Von Neumann’s architecture. Very dense architectures for 3D-integration of CMOS computing units with crossbar arrays of nanodevices like the semiconductor/nanowire/molecular integrated circuits (CMOL) [147] architecture have been proposed. A CMOL system combines the advantages of CMOS technology (flexibility and high fabrication yield) with the high density of crossbar arrays of nanoscale devices. This structure consists of a dense nanowire crossbar fabric on top of the CMOS substrate with memristor devices assembled in the crossings between nanowires as shown in Figure 8. Figure 8a shows a crossbar nanoarray where nanowires run in orthogonal directions. A memristive device is located at each cross point of a vertical and horizontal nanowire. Figure 8b shows the proposed CMOL structure. The nanowire crossbar is tilted with respect to the orientation of the 2D array of CMOS neurons. Each CMOS neuron has an output pin (red dots in Figure 8b) and an input pin (blue dots in Figure 8b). Each neuron output is connected to just one nanowire and each neuron input is connected to another nanowire in the perpendicular direction. The crosspoint memristive devices implement the synaptic connections between neurons. In the illustration of Figure 8b, the output of neuron 2 is connected to the input of neuron 1 through the synaptic memristive device located at the intersection point (marked as a black circle) of the two perpendicular nanowires (plotted as green lines) connected to neuron 2 output and neuron 1 input, respectively. Other alternative architectures for neuromorphic structures based on 3D integration of CMOS neurons and memristive synapses have been proposed as CrossNets [148]. A functional digital FPGA-like implementation of a small CMOL prototype where the memristors where used as digital switches to re-configure the digital hardware implemented in the CMOS cells has been demonstrated [149].

Neuromorphic architectures composed of CMOS neurons connected to a crossbar array of memristors as shown in Figure 8c have also been proposed as accelerators to perform the intensive matrix multiplications needed in deep machine learning architectures. In the memristive crossbar shown in Figure 8c, the input vector $\left[V_{in1},V_{in2},\ldots ,V_{inN}\right]$ is applied as input voltages to the rows, each memristor in an (i,j) crossbar position is programmed with an analog value $w_{ij}$ so that the currents flowing through the vertical columns are the result of the vector-matrix multiplication

(4) $$\begin{array}{c} I_{j}=\sum w_{ij}V_{ini}. \end{array}$$

Many works have proposed including ReRAM memristive memory crossbars to implement Matrix-Vector-Multiplication Units in computer architectures to accelerate Neural Network applications [150,151,152,153,154,155] demonstrating great benefits in power consumption levels. PRIME [151] and RESPARC [150] report simulations of energy savings compared to fully CMOS Neural Processors Units in the order of $10^{3}$ depending on the particular neural network architecture. Energy savings in the order of 10${}^{3}$–10${}^{5}$ respect to baseline CPU implementations have been reported [153,155]. However, in these works the memristor crossbars are included at a simulation level. A real hardware implementation of a hybrid CMOS system including an array of ReRAM crossbar as vector matrix multiplication elements for neural network computing acceleration at low energy consumption has been reported [22]. However, in this work the memristors are used in digital flip-flops as non-volatile digital devices. The real integration of CMOS neurons with a crossbar of CBRAM memristors is also demonstrated [156] for functional programming of a crossbar array of memristors in a digital way. More advanced fabrication techniques have been proposed to integrate up to 5 layers of 100 nm memristors in 3D crossbar arrays [157]. Some works have demonstrated the feasibility of integrating both carbon nanotube field-effect transistors (CNFETs) and RRAM on vertically stacked layers in a single chip on top of silicon logic circuitry, reporting 1952 CNFETs integrated with 224 RRAM cells for brain-inspired computing [158], or a prototype with more than 1 million RRAM cells with more than 2 million CNFETs in a single chip [25]. A recent work reported some circuit-level techniques for the design of a 65 nm 1 Mb pseudo-binary nonvolatile computing-in-memory RRAM macro which is capable of storing 512 k weights for Deep Neural Networks (DNN) [159].

However, so far experimental demonstrations of classification and training of memristive based analogue-memory learning systems have been on reduced systems and without achieving monolithic integration of the CMOS and memristive part [160], and suffered from classification inaccuracies due to device imperfections as control of the weight update, the programming of multilevel values, or variation in the device conductance range, limiting their application and severely degrading the performance of the network [161,162]. Another important shortcoming that limits the density of the implemented crossbars, as well as the practical hardware implementation of CMOL neuromorphic memristive systems, is the necessity of implementing a MOSFET in series with each memristive device (the so-called 1T1R devices) to limit the currents flowing through each memristor avoiding damage due to transient high-currents. When the transistor device is omitted, the current limitation is done in the peripheral CMOS circuitry, limiting the size of the array to reduce the risk of local high parasitic transient currents. In the 1T1R structures, the transistor also acts as a selection device to update individually each memristor avoiding alteration of the nearby devices. As a summary, although memristors are a very promising technology to implement high-density analog memories close to the computing system that could potentially implement high-speed low power learning cognitive system, there are still some technological limitations that are currently being investigated that have not allowed to implement such large scale systems.

## 5. Learning with Memristors (STDP)

Given that these SNNs are more powerful, in theory, than 2nd generation networks, it is natural to wonder why we do not see widespread use of them. One main issue that currently lies in practical use of SNNs is that of training. Learning mechanisms are crucial for the ability of neuromorphic systems to adapt to specific applications. In general, the goal of a learning algorithm is to modify the weights of the synaptic connections between neurons in order to improve the response of the network to a certain stimulus. Two main categories can be considered: supervised or unsupervised learning. In supervised learning, the dataset samples are labeled with the identification of the expected ‘correct’ network output. The measured deviation between the desired output and the real one is used to modify the synaptic weights. In unsupervised learning, there is no labeled information, so the own characteristics of the input data are analyzed by the network in order to self-organize.

As explained in Section 2, in the ANN field, the powerful computational capabilities of modern GPUs and CPUs and the availability of large amount of annotated data have made possible to train complex deep learning architectures using the supervised backpropagation learning algorithm [48] to solve complex cognitive problems in some cases with better accuracy than humans. However, there are no known effective supervised training methods for SNNs that offer higher performance than 2nd generation networks. The popular backpropagation learning strategies are not directly usable in SNN networks. On the one hand, if spikes are represented computationally as the occurrence of an output event at a particular time (as represented in Figure 3) they are not differentiable; on the other hand, differentiating the error back across the spatial layers (as it is done in the backpropagation algorithm) looses the precise temporal information contained in the spike timings. Therefore, in order to properly use SNNs for real-world tasks, we would need to develop an effective supervised learning method that takes space and time simultaneously into account [163]. Several approaches for SNN training have been adopted:

**Training an ANN and conversion to SNN** [66,164,165,166,167]. Some authors have proposed ANN to SNN direct conversion methods which are based on the training of ANN using static input images and directly mapping the network to an SNN converting the input stimulus to spikes using frequency rate encoding [164,165,167]. Bodo et al. implemented several optimizations achieving for a rate coded input similar performance than equivalent ANN implementations [165]. However, such encoding reduces the power efficiency of SNN. Other authors have proposed to train SNN with sensory data coming directly from a spike-based sensor (as a DVS recording). For that purpose, an equivalent ANN using static images generated from histograms of the input recordings of spiking stimulus is trained. Afterwards, a method to convert the weights of the ANN to the corresponding SNN is devised [66]. The additional timing parameters as leakage time or refractory period characteristics of SNN are optimized as hyper-parameters in the SNN resulting on different optimized parameter values for different input dynamics. Bodo et al. recently proposed an ANN to SNN conversion method based on time-to-first-spike input conversion code [166]. In all of these methods, training is done on static images and thus they do not fully exploit directly all the spatio-temporal information contained in the events.

**Supervised training in the spiking domain**. For the above mentioned reason, some methods for direct supervised learning in the spiking domain have been proposed [168,169,170,171,172,173,174,175,176,177,178,179]. Some of the earlier SNN training methods were based on an adaptation of the Delta Learning Rule [44] and were appropriate to train single layer architectures [169,171,172]. More recent SNN learning methods have been reported that try to apply the backpropagation learning rules to SNN with several learning layers. They include coding the spike times to have a differentiable relationship with a subset of previous spikes and hence compatible with the gradient descent back-propagation rule in the temporal domain [180], or approximating the spike shape response activity to be differentiable across neural layers [174,175,177]. Wu et al. introduced an SNN Spatio-Temporal BackPropagation algorithm [177]. Not only do they approximate the spike shape as a continuous differentiable function, but also they use a back-propagation-through-time (BTT) [163] which backpropagates the error in the space as well as the time dimension reporting the best recognition accuracy achieved by previously reported SNN on the MNIST and N-MNIST datasets and equivalent to the state-of-the-art of ANNs. Similarly, the SLAYER method [178] considers back-propagation in space and time and trains both weights and delays of the synaptic connections.

**Figure 9:**
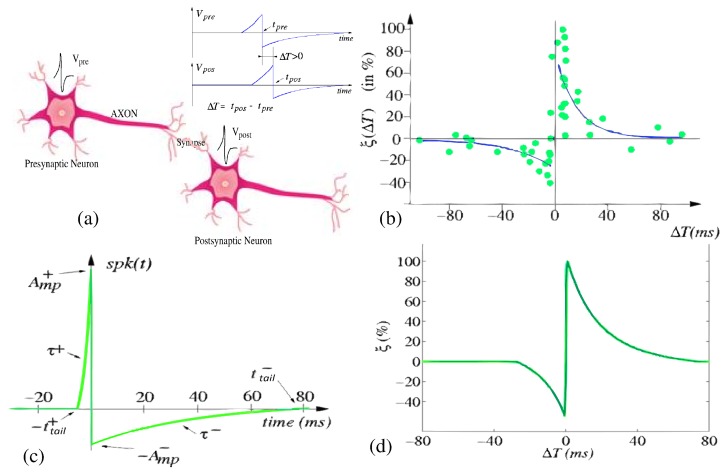
Illustration of STDP learning rule. (**a**) Pre-synaptic neuron generating a spike $V_{pre}$ at time $t_{pre}$ that arrives to a post-synaptic neuron that generates a spike $V_{post}$ at time $t_{post}$, being $\Delta T=t_{post}-t_{pre}$, and (**b**) illustrates the variation of the synaptic efficacy ξ(ΔT) Vs ΔT, STDP learning rule, as the observed by Bi and Poo in biological synapses. (**c**) Illustrates the spike shape that applied to the memristive devices describes in Section 4 reproduces the STDP learning rule shown in (**d**).

**Unsupervised training in the spiking domain**. The unsupervised SNN training methods are mostly based on the well known Spike-Timing-Dependent Plasticity (STDP) learning rule [31,32]. STDP is a Hebbian learning rule. The traditional Hebbian synaptic plasticity rule was formulated in 1940 suggesting that synapses increase their efficiency if they persistently take part in firing the post-synaptic neuron [39]. Much later in 1993, STDP learning algorithms were reported [31,32] as a refinement of this rule taking into account the precise relative timing of individual pre- and post-synaptic spikes, and not their average rates over time. In comparison with traditional Hebbian correlation-based plasticity, STDP proved to be better suited for explaining brain cortical phenomena [181,182], and demonstrated to be successful in learning hidden spiking patterns [183] or performing competitive spike pattern learning [184]. Interestingly, shortly after that, in 1997, STDP learning was experimentally observed in biological neurons [33,34,35]. Figure 9a,b illustrate the STDP learning rule as observed in biological synapses. Figure 9a plots a presynaptic neuron with a membrane potential $V_{pre}$ which is connected through a synapse with synaptic strength *w* to a postsynaptic neuron with membrane potential $V_{post}$. The presynaptic neuron emits a spike at time $t_{pre}$ which contributes to the generation of a postsynaptic spike at time $t_{post}$. The biological learning rule observed by Bi and Poo is illustrated in Figure 9b. When the two connected neurons generate spikes close in time, if $\Delta T=t_{post}-t_{pre}$ is positive, meaning that the presynaptic pulse contributed causally to generate the postsynaptic pulse, there is a positive variation in the efficacy of the synaptic connection ξ(ΔT)>0; on the contrary, if $\Delta T=t_{post}-t_{pre}$ is negative, the variation in the efficacy of the synaptic connection ξ(ΔT)<0 is negative. Being STDP a local learning rule, and memristors two-terminal devices exhibiting plasticity controlled by the local applied voltage/current to their terminals converts memristors as ideal candidates to implement high-density on-line STDP-based neuromorphic learning systems [27]. Linares et al. [28] showed that by combining the memristance model formulated in Equation (2) with the electrical wave signals of neural impulses (spikes) as shaped in Figure 9c applied to the pre- and post-synaptic terminals of the memristive synaptic-like device, the STDP behavior shown in Figure 9d emerges naturally. Considering the mathematical equation describing the spike shape shown in Figure 9c versus time

(5) $$\begin{array}{c} spk\left(t\right)=\left\{\begin{array}{cc} A_{mp}^{+}\frac{e^{t/\tau^{+}}-e^{t_{tail}^{+}/\tau^{+}}}{1-e^{t_{tail}^{+}/\tau^{+}}} & if\;-t_{tail}^{+}<t<0\\ A_{mp}^{-}\frac{e^{-t/\tau^{-}}-e^{-t_{tail}^{-}/\tau^{-}}}{1-e^{-t_{tail}^{-}/\tau^{-}}} & if\;0<t<t_{tail}^{-}\\ 0 & otherwise \end{array}\right. \end{array}$$

and a memristive synapse-like device where a presynaptic spike spk(t) with attenuation $\alpha_{pre}$ arrives at time *t* to its negative terminal and a postsynaptic spike spk(t+ΔT) with attenuation $\alpha_{pos}$ arrives at time t+ΔT to its positive terminal, a voltage difference

(6) $$\begin{array}{c} v_{MR}\left(t+\Delta T\right)=\alpha_{pos}spk\left(t+\Delta T\right)-\alpha_{pre}spk\left(t\right) \end{array}$$

is generated among the device terminals. The total change in the memristance parameter *w* can thus be computed as,

(7) $$\begin{array}{c} \Delta w\left(\Delta T\right)=\int f_{MR}\left(v_{MR}\left(t+\Delta T\right)\right)dt=\xi \left(\Delta T\right) \end{array}$$

Interestingly, for the memristor model considered in Equation (2) and the spike shape considered in Equation (5), the memristance learning rule shown in Figure 9d ξ(ΔT) is obtained which resembles the STDP rule observed by Gerstner in biological neurons. By playing with the spike shapes, many other STDP update rules can be tuned as demonstrated by Zamarreño et al. [29,30].

In the last decade, many different works have demonstrated the emergence of STDP learning in memristive devices of different kinds of materials [137,180,185,186,187,188,189]. However, as already stated in Section 4, at a system level, the current limitations of the memristor technology in terms of control of the resolution of the weigh updating, have not made possible the implementation of working STDP memristive learning systems with analog synaptic elements. Precision in the weight update is difficult to control and most of the memristive devices operate changing between binary states. For that reason, stochastic STDP learning rules that operate with binary weights during inference and updating operation have been proposed. Seo et al. [190] applied this idea to simple classification problems, but they found that they could not learn to separate more than 5 patterns. Recently, Yousefzadeh et al. [191] were able to classify more elaborated databases (as MNIST) by introducing some other techniques that improved the performance.

**Combining unsupervised feature extraction methods with supervised categorization training**. While supervised learning methods like backpropagation are not energy efficient, are not appropriate for on-line chip learning, and do not look like biologically plausible, unsupervised learning rules are appropriate to extract repetitive structures in the training data but not appropriate to take decisions [192,193]. For example, Mozafari et al. propose to combine unsupervised STDP layer with supervised Reinforcement Learning STDP layers [193]. The resulting network is more robust to overfitting compared to backprogation training as it extracts common features and performs well with reduced number of training samples.

## 6. Future Perspective

It is well known that the human brain contains about $10^{11}$ neurons interconnected through $10^{15}$ synapses, and with a power consumption of around 20 W it is capable of performing complex sensing and cognitive processing, sophisticated motor control, learning and abstraction, and it can dynamically adapt to changing environments and unpredicted conditions. For this reason, neuromorphic engineers have been using the brain as a processing paradigm for several decades in order to fabricate artificial processing systems with similar capabilities. After the initial attempts of building the first spike-based processing systems demonstrated their feasibility and showed their promising potential [78], it became evident the need for scaling up these systems in terms of number of neurons and synapses [62]. Several works developed by both academic institutions [86,121,122,123,124,125,127,128,129] and industrial players like IBM [120] or Intel [126] fabricated neuromorphic chips with up to 1 M neurons and 256 M synapses, which could be ensembled in multi-chip boards and multi-board platforms, opening the way to implement large systems in the near future with numbers of neurons and synapses similar to the brain. However, these systems, based on different CMOS technologies, will be limited by the their large room-scale size. Besides, the complexity of current implementations of learning algorithms in CMOS limits their scalability.

The emergence of memristors and their synaptic-like behavior opened the possibility to overcome the limitations of CMOS technologies. Memristors can be a few nanometers size and can be packed densely in a two-dimensional layer with nanometer-range pitch, potentially offering higher neuron and synaptic density. With a fabrication process much cheaper than CMOS, memristor layers can be stacked in 3D. Assuming a reasonable 30-nm pitch, the superposition of 10 memristive layers could theoretically provide a memory density of $10^{11}$ non-volatile analog cells per cm${}^{2}$. This approach could in principle reach the neuron and synaptic density of the human brain in a single board, including learning capabilities [194]. Furthermore, the close 3D dense packaging between the CMOS neural computation units and the memristive adaptive memory synaptic elements can significantly reduce the current consumption of the resulting systems.

Current available memristors are described as 1T1R devices, meaning that they are formed by the series connection of a MOS transistor and a memristive element. This transistor is used to limit the current flowing through the memristor during each operation (Forming, Writing, Erasing, Reading) to avoid damaging the device. However, this structure is limiting the density of memristors, as they are also consuming area in the CMOS substrate. An alternative to overcome this limitation is given by 1S1R devices (1-selector-1-resistor), where a volatile memristor (1S) is connected in series with a non-volatile memristor (1R), eluding any CMOS area consumption [195].

Hybrid systems with memristor layers fabricated on top of a CMOS substrate can provide highly parallel massive storage tightly coupled to CMOS computing circuitry. Therefore, computing and learning processes in the brain can be imitated by combining memristors with spiking processors and integrate-and-fire neurons in silicon. Using mesh techniques [82], grids of tens of chips can be assembled modularly on a Printed Circuit Board (PCB), allowing for scaling up the numbers of neurons and synapses in a neural system [65]. The combination of all these techniques together with the resolution of the multiple technical challenges currently associated to dense memristive layers (reliability, repeatability, reprogrammability) could provide an important step towards the hardware implementation of brain-scale low-power neuromorphic processing systems with online STDP learning.

## Figures and Tables

**Table 1:** Table comparing different features of ANNs and SNNs.

Feature	ANN	SNN
Data processing	Frame-based	Spike-based
Latency	High	Low
		Pseudo-simultaneity
Time resolution	Low	High
		Preservation of spatio-temporal
		correlation
Time processing	Sampled	Continuous
Neuron model complexity	Low	High
Recognition accuracy	Higher	Lower
Hardware multiplexing	Possible	Not possible
System scale-up	Ad hoc	Adding modules
Recognition speed	Low	High
	Independent on input stimulus	Dependent on input statistics
	Dependent on hardware resources	
	Dependent on system complexity	Not dependent on system complexity
Power consumption	Determined by processor power	Determined by power-per-event
	and memory fetching	processing in modules
	Independent on input stimulus	Dependent on stimulus statistics
Recurrent topologies	Need to iterate until converge	Instantaneous

**Table 2:** Comparison of the major features of the human brain and the large-scale neuromorphic systems described in this work.

Platform	Human Brain	Neurogrid	BrainScaleS	Truenorth	SpiNNaker	Loihi	Darwin	ROLLS	DYNAPs	ODIN
Technology	Biology	Analog, sub-threshold	Analog, over threshold	Digital, fixed	Digital, programmable	Digital, programmable	Digital, programmable	Mixed-signal, sub-threshold	Mixed-signal, subthreshold	Digital, programamble
Feature size	10 μm	180 nm	180 nm	28 nm	130 nm	14 nm	180 nm	180 nm	180 nm	28 nm
# transistors		23 M	15 M	5.4 B	100 M	2.07 B	≈M	12.2 M	-	-
Chip size		1.7 cm${}^{2}$	0.5 cm${}^{2}$	4.3 cm${}^{2}$	1 cm${}^{2}$	60 mm${}^{2}$	25 mm${}^{2}$	51.4 mm${}^{2}$	43.79 mm${}^{2}$	0.086 mm${}^{2}$
# neurons (chip)		65 k	512	1 M	16 k	131 k	≈M	256	1 k	256
# synapses (chip)		100 M	100 k	256 M	16 M	126 M	Programmable	128 k	64 k	64 k
# chips per board		16	352	16	48	-	-	-	-	-
# neurons (board)	$10^{11}$	1 M	200 k	16 M	768 k	-	-	-	-	-
# synapses (board)	$10^{15}$	4 B	40 M	4 B	768 M	-	-	-	-	-
Energy per connection	10 fJ	100 pJ	100 pJ	25 pJ	10 nJ	81 pJ	10 nJ	>77 fJ	30 pJ	12.7 pJ
On-chip learning	Yes	No	Yes	No	Yes	Yes	Yes	Yes	No	Yes
